# Effects of the Use of N95 Masks on the Vital Signs of Healthy Healthcare Workers During the COVID-19 Pandemic: A Hospital-Based Cross-Sectional Study

**DOI:** 10.7759/cureus.40622

**Published:** 2023-06-19

**Authors:** Pradeep Kumar, Kunal Nath, Arun Prasad, Lokesh K Tiwari, Bhabesh K Chowdhry, Amit K Sinha, Neha Chaudhary

**Affiliations:** 1 Pediatrics, All India Institute of Medical Sciences, Patna, IND; 2 Pediatrics, All India Institute of Medical Sciences, Rishikesh, IND; 3 Neonatology, All India Institute of Medical Sciences, Patna, IND; 4 Pediatric Surgery, All India Institute of Medical Sciences, Patna, IND; 5 Community and Family Medicine, All India Institute of Medical Sciences, Patna, IND

**Keywords:** healthcare workers, droplets, coronavirus, personal protective equipment, n95 filtering facepiece respirators

## Abstract

Background and aims: The N95 filtering facepiece respirator (FR) is the most commonly recommended respiratory protection used in healthcare settings. However, concerns have been raised about its use because it can increase respiratory resistance and dead space. The primary objective of this study was to determine the effect of wearing N95 masks on the vital signs, i.e., oxygen saturation, pulse rate, and respiratory rate, of the participant health workers. Our secondary objective was to assess the subjective feeling of discomfort when wearing N95 masks.

Methods: The study participants were healthy healthcare workers taking care of coronavirus disease 2019 (COVID-19)-infected pediatric cases who did at least six hours of continuous shift duty in the pediatric COVID-19 ward at a tertiary care hospital in the eastern part of India. They were evaluated for vital signs at various time intervals while wearing N95 masks. Subjective discomfort at any point in time was also noted.

Results: We found a significant variation in the mean oxygen saturation (SpO_2_) and heart rate (HR) reduction across the four different points. The pair-wise comparison showed a small but significant decrease in the mean SpO_2_ of 98.3% (1.1) at six hours as compared with a mean SpO_2_ of 98.7% (0.9) at three hours. Similarly, a significant increase was noted for a mean HR of 84.7 bpm (11.2) at six hours compared with a baseline of 82.3 bpm (9.2) and 83.2 bpm (8.8) at three hours.

Conclusion: The continuous use of an N95 mask leads to a mild increase in respiratory rate. However, heart rate and oxygen saturation vary significantly at different points in time after N95 mask use.

## Introduction

India registered the first coronavirus disease 2019 (COVID-19) case in Kerala on January 30, 2020. On March 11, 2020, the World Health Organization declared COVID-19 a global pandemic [1]. In the case of a pandemic involving an airborne-transmissible agent, the use of a mask for protection is a must in healthcare settings [2].

Activities like walking slowly during rounds with an N95 mask are found to increase inhaled carbon dioxide, reduce inspired oxygen, and increase the work of breathing. This results in inhaled carbon dioxide of 2% to 3% (normal range: 0.04%), which produces transient acidosis and a compensatory increase in minute ventilation, work of breathing, and cardiac output. This leads to increased sweating, headaches, visual changes, breathing difficulty, enhanced irritability, lowered reasoning capacity, alertness, and exercise tolerance. Independently, the inspired oxygen of 17% (normally 21%) leads to headache, light-headedness, drowsiness, muscle weakness, dyspnea on exertion, nausea, and vomiting. Simultaneously, the augmented resistance to inspiratory (15% of maximum) and expiratory flow for more than 10 minutes results in respiratory alkalosis, fatigue, increased lactate levels, and decreased physical work capacity [3-5]. Increased breathing resistance with a respirator results in a shift to anaerobic metabolism due to an increased rate of oxygen (O_2_) debt and early exhaustion at lighter workloads [4]. Increased breath resistance augments the work of respiration via the additional physiological burden of compensatory mechanisms. Breathing resistance with respirators has been identified as the cause of respiratory fatigue and impaired physical work capacity due to a shift to anaerobic metabolism from an increased rate of O_2_ debt. However, the study on the breathing resistance of respirators is still in its infancy [6].

There is conflict over current recommendations regarding the protection of healthcare workers against COVID-19 in non-aerosol-generating situations [7]. Individual respiratory protective devices and face masks represent critical tools in protecting healthcare workers in hospitals and clinics and decreasing the spread of highly infectious COVID-19. These recommendations aim to reduce respiratory droplet excretion in pre-symptomatic and asymptomatic individuals (source control) [8].

Despite widespread use, few published data exist regarding the physiological impact of filtering facepiece respirators (FFRs) on healthcare workers. We assessed the physiological impact on healthcare workers due to the use of an N95 FFR mask with and without an exhalation valve.

## Materials and methods

This hospital-based cross-sectional study was done at the pediatric COVID-19 ward of the All India Institute of Medical Sciences, Patna, India, from August through October 2020. The study was conducted with the approval of the Institutional Ethics Committee of the All India Institute of Medical Sciences, Patna, India (reference number: AIIMS/Pat/IEC/2020/536).

The study’s primary objective was to study the effect of wearing N95 masks on vital signs, i.e., oxygen saturation, pulse rate, and respiratory rate. The secondary objective was to assess the physiological effects of wearing N95 masks subjectively in the form of headache, restlessness, dizziness, fatigue, perspiration, air hunger or breathlessness, and pressure discomfort. The hospital was declared a dedicated COVID-19 hospital in the first wave of the pandemic, treating only COVID-19 patients. All healthy healthcare workers, including resident doctors and nursing officers taking care of suspected and COVID-19-infected cases with at least six hours of continuous shift duty (the night shift was not included to avoid the circadian variability affecting the results of our study), were included in the study as participants. They had to wear personal protective equipment (PPE), including N95 masks, throughout their shift without taking anything off in between, in compliance with standard operating procedures (SOPs) and guidelines issued by the hospital administration regarding the management of COVID-19 patients. Pregnant healthcare workers, chronic smokers, and people with chronic obstructive pulmonary disease (COPD) and significant cardiorespiratory illnesses were excluded from the study.

Healthcare workers fitting the inclusion criteria were well-informed about the nature and objective of the study. After taking proper oral and written consent, variables such as oxygen saturation, respiratory rate, and heart rate were measured at zero, one, three, and six hours of their shift duty each time. Zero hours implied the time at which donning the mask was performed. In our study, N95 masks were included both with and without expiration valves due to time-to-time availability, as government procurements go through set rules.

The baseline blood pressure and temperature of participants were taken before donning personal protective equipment (PPE), but they were not included in the study given the inability to measure them while wearing PPE.

For each participant, basic details such as age, designation, sex, weight, height, body mass index, blood pressure, body temperature, health concerns, and medication history were recorded separately. Any subjective discomfort perceived, such as headache, restlessness, dizziness, fatigue, perspiration, air hunger or breathlessness, and pressure discomfort, was ascertained. The psychological effects of continuous use of N95 masks and the psychological status of participants enrolled in the study were not taken into account.

The pulse oximeter used was the Signature Series fingertip pulse oximeter (201, Dr. TrustTM, USA), with an audio-visual alarm on the right hand’s middle finger (to ensure uniformity). Respiratory rate was measured manually over one minute, while pulse rate was measured over one minute at the wrist by using the index and middle fingers, which was in accordance with the readings shown on the pulse oximeter.

We used N95 masks manufactured by Venus Safety and Health Limited, Magnum Health and Safety Private Limited, and the Defence Research and Development Organization (DRDO) India. Masks were National Institute for Occupational Safety and Health (NIOSH) and/or Bureau of Indian Standards (BIS)-approved. The shapes of the masks were cup-shaped, duck bill-shaped, V-shaped, C-shaped, or flat-folded, depending upon availability in each shift for participant healthcare workers.

All the data underwent distribution analysis using the Shapiro-Wilk test first. Then all continuous values were expressed as means and standard deviations, whereas categorical data were presented as the median and interquartile range (IQR). For normally distributed data, comparisons were made using unpaired t-tests and a one-way repeated measures analysis of variance (ANOVA). The Mann-Whitney U test was used to compare data that were not normally distributed. The Chi-square test was applied to determine the association between categorical variables. The significance level was defined as p < 0.05. The data were analyzed using Stata version 13 software.

## Results

A total of 91 healthcare workers participated in the study, depending on the duty roster in our COVID-19 pediatric ward. This sample size was reached based on the participation of healthcare workers from August to October 2020. The median body mass index (BMI) for each gender was approximately 23.5 kg/m2. Maximum participants were working as nursing officers, i.e., male nursing officers (27 (60%)) and female nursing officers (37 (80.4%)), followed by nine junior residents (20%) in the male group and four senior residents (8.7%) in the female group (p-value = 0.03) (Table 1).

**Table 1:** General characteristics of the study participants *Statistically significant SD: standard deviation; IQR: interquartile range; BMI body mass index

Characteristics	Gender		p-value (test of significance)
	Male	Female	
Age: Mean ± SD (95% CI)	27 ± 2.9 (26.2- 27.9)	28.2 ± 3.5 (27.2- 29.3)	0.04*
BMI: Median (IQR)	23.6 (8.9)	23.4 (10)	0.423
Designation: n (%)	45 (100)	46 (100)	0.03*
Junior resident n (%)	9 (20)	2 (4.4)	
Nursing officer n (%)	27 (60)	37 (80.4)	
Senior nursing officer n (%)	1 (2.2)	3 (6.5)	
Senior resident n (%)	8 (17.8)	4 (8.7)	

The mean value of physiological parameters was noted consecutively at baseline, then after one hour, followed by three hours, and finally at six hours (Table 2).

**Table 2:** Repeated measure ANOVA comparing physiological parameters at four different time points for the participants after using the N95 mask SD: standard deviation; df: degree of freedom; * p-value is significant

Physiological parameters	Baseline mean (SD)	One-hour mean (SD)	Three-hour mean (SD)	Six-hour mean (SD)	F test (df), p-value
Heart rate	82.3 (9.2)	82.1 (9.9)	83.2 (8.8)	84.7 (11.2)	4.4 (2.6- 238.9), 0.005*
Respiratory rate	19.3 (2.2)	19.4 (2.4)	19.7 (2.3)	20.1 (3.8)	2. 7 (2.1-185), 0.07
Oxygen saturation	98.6 (0.9)	98.6 (0.9)	98.7 (0.9)	98.3 (1.1)	2.8 (2.6, 236.3), 0.04*

A repeated-measure one-way ANOVA test was applied, which revealed a significant variation of oxygen saturation (SpO2) (p-value = 0.04) and heart rate (HR) (p-value = 0.005) across these four different time points after wearing an N95 mask (Table 3).

**Table 3:** Pairwise comparison of SpO2 and heart rate over various time points with LSD correction SpO2: oxygen saturation; *CI: confidence interval; LSD: least significant difference; *p-value is significant

Pair	Mean difference in SpO_2 _(95% CI), p-value	Mean difference in heart rate (95% CI), p-value
Baseline: 1 hour	-0.03 (-0.26- 0.19) 0.77	0.26 (- 1.5- 2.1) 0.77
Baseline: 3 hours	-0.16 (-0.38- 0.05) 0.14	-0.99 (- 2.6 - 0.66) 0.23
Baseline: 6 hours	0.19 (-0.84- 0.48) 0.166	-2.4 (- 4.40- 0.52) <0.01*
1 hour to 3 hours	-0.13 (-0.32- 0.06) 0.181	-1.2 (-2.6- 0.17) 0.08
1 hour to 6 hours	-0.23 (-0.04- 0.5) 0.102	-2.7 (- 4.1 - -1.3) <0.001*
3 hours to 6 hours	0.36 (0.08- 0.64) 0.01*	-1.4 (- 2.9- 0.08) 0.063

Pairwise comparison using least significant difference (LSD) correction showed a small but significant decrease in mean SpO2 at three hours versus six hours (98.7 (0.9) versus 98.3 (1.1)). On the contrary, for mean HR, significant increases were noted at baseline versus six hours and at one hour versus six hours (82.3 (9.2) vs. 84.7 (11.2)) and (82.1 (9.9) vs. 84.7 (11.2)), respectively. However, no significant variation or non-significant mild increase was observed for the mean respiratory rate over four different time points. Restlessness was the complaint of only five (5.5%) participants-three (6.7%) males and two (4.3%) females. Only six (6.6%) participants-five (11.1%) males and one (2.2%) female-experienced air hunger. Perspiration was felt by 13 (14.3%) participants-four (8.9%) males and nine (19.6%) females. Also, only six (6.6%) participants mentioned feeling fatigued-four (8.9%) males and two (4.4%) females. Furthermore, 16 (17.6%) participants-eight (17.8%) males and eight (17.4%) females-reported experiencing pressure discomfort after wearing an N95 mask. However, dizziness was not a complaint by any of the participants (Table 4).

**Table 4:** Adverse effects experienced by participants after using the N95 mask * p-value of both males and females together

Adverse effects		Gender		p-value (Chi-square test)*
		Male (%)	Female (%)	
Restlessness	Yes	3 (6.7)	2 (4.3)	0.627
	No	42 (93.3)	44 (95.7)	
Dizziness	Yes	0 (0)	0(0)	-
	No	45 (100)	46 (100)	
Air hunger	Yes	5 (11.1)	1 (2.2)	0.08
	No	40 (88.9)	45 (97.8)	
Perspiration	Yes	4 (8.9)	9 (19.6)	0.146
	No	41 (91.1)	37 (80.4)	
Pressure discomfort	Yes	8 (17.8)	8 (17.4)	0.96
	No	37 (82.2)	38 (82.6)	
Fatigue	Yes	4 (8.9)	2 (4.4)	0.38
	No	41 (91.1)	44 (95.6)	

**Figure 1:**
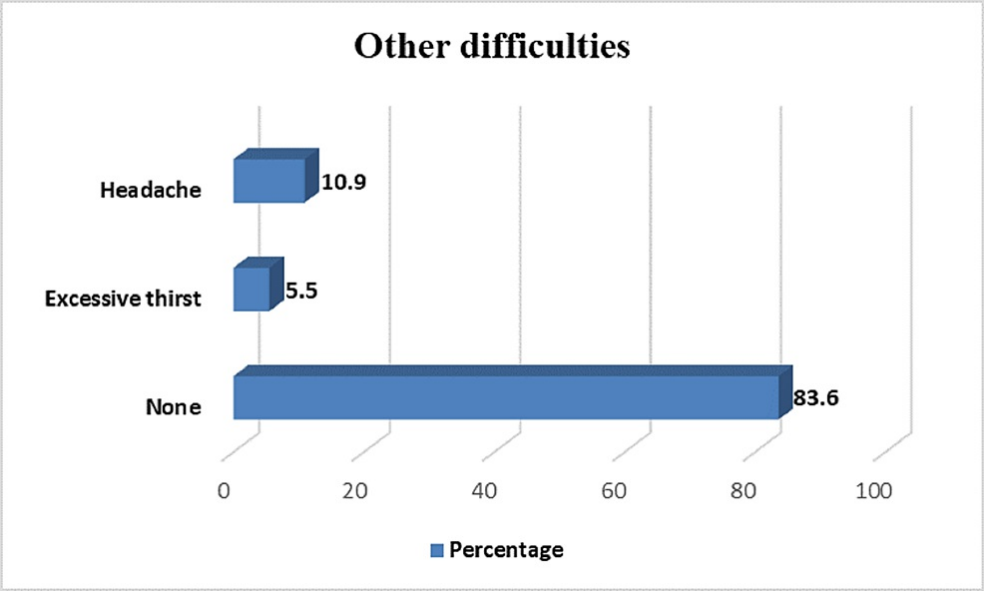
Other difficulties experienced by participants after using the N95 mask

As far as any other difficulties experienced by the participants were concerned, only 10 (10.9%) and five (5.5%) reported headaches and excessive thirst (Figure 1).

## Discussion

Our study found a nonsignificant small increase in respiratory rate while wearing N95 masks for a prolonged duration. This is contrary to most of the literature available. In a study done by Kim et al., they found that wearing an N95 mask for one hour at a low-moderate workload may result in an increased respiratory rate as compared to controls [9]. Also, Harber et al. found in their study that increased dead space inside the N95 mask may cause increased tidal volume and an increased respiratory rate [10]. Bansal et al. found that wearing a respirator while performing tasks requiring moderate exertion caused increased inspiratory tidal volume, minute ventilation, respiratory rate, heart rate, and duty cycle [11]. Lim et al. also found a significantly increased respiratory rate among the N95 mask wearers at four hours [12]. These findings were contrary to the study done by Fikenzer et al., who found a significant reduction in breathing frequency [8]. Similarly, Roberge et al. found a non-significant mild decrease in respiratory rate with the use of N95 FFR masks for one hour [13].

Our study shows a small but significant increase in heart rate at baseline versus six hours of duration and also at one hour versus six hours of duration. The clinical effects of these changes were not observed. In a study done by Kim et al., compared with controls, respirator use was associated with mean one-hour increases in heart rate (range: 5.7-10.6 beats per minute, p< 0.001), respiratory rate (range: 1.4-2.4 breaths per minute, p< 0.05), and transcutaneous carbon dioxide (range: 1.7-3.0 mm Hg, p< 0.001). However, no significant differences in oxygen saturation between controls and respirators were noted (p>0.05) [8]. In their study, Li et al. found that heart rate, microclimate temperature, humidity, and skin temperature inside the facemask, together with perceived humidity, heat, breathing resistance in the facemask, itchiness, fatigue, and overall discomfort, were significantly higher (p<0.01) for N95 masks than for surgical masks. [14] On the contrary, Fikenzar et al., Roberge et al., Tong et al., and Harber et al., in their respective studies, found minimal or no variation in heart rate upon the use of N95 among their chosen study population [8,10,13,15].

Oxygen saturation was significantly reduced at three hours versus six hours, but no episode of hypoxia was noticed clinically. However, the changes were statistically more significant than clinical. Similarly, Roberge et al., while measuring the ear and fingertip oxygen saturation of healthcare workers wearing protective masks, found significant differences in SpO_2_ values, but the absolute differences were small and no episodes of hypoxia were observed [13]. Laferty et al. found that oxygen saturation changed only < 1% during qualitative respirator fit testing. The study done on N95 masks in pregnant healthcare workers also shows similar results [16]. Fukushi et al. conducted experiments in which participants performed a progressive treadmill exercise and had no harmful effect on oxygen saturation during exercise at any load level [17]. In a study by Rehman et al., they found no significant difference in oxygen saturation in the staff of the oral dental department after prolonged wearing of a filtering facepiece 3 (FFP3) mask [18]. In healthy young men (age 25.7 ± 3.5 years), in a study by Lassing et al., the use of surgical face masks was associated with reduced oxygen uptake during continuous exercise [19]. Shein et al. found that facemasks did not impair oxygenation or ventilation among 50 adults at rest or during physical activity [20].

In our study, subjective discomfort by the wearers of the N95 mask was also ascertained, ensuring adequate compliance with wearing it. Pressure discomfort caused by the strap was complained about by 16 participants (17.6%). Perspiration in excess was felt by 13 participants. It was followed by air hunger and fatigue, complained of by six participants. Five participants experienced restlessness. Among other complaints, headaches, and excessive thirst were complained of by 10 and five participants, respectively. In a study done by Lim et al., 37% of healthcare workers surveyed reported headaches following FFR use. They link this side effect to the rebreathing of inhaled CO_2_ inside the respirator microenvironment. The response to this environment is an increased rate and depth of breathing and cardiac output [12]. Roberge et al. found that continuous use of an N95 mask that exceeded four hours was associated with the development of headaches [13]. While pressure discomfort can be attributed to the nature of the material used in the N95 mask and can depend upon the N95 mask type, there are several explanations regarding other side effects. Exposure to inhaled CO_2_ between 2% and 3% can cause sweating, headaches, and dyspnea, which can be felt by some subjects at rest for several hours [15]. If concentration reaches 4-5%, dyspnea can occur within several minutes, and increased BP, dizziness, and headaches can occur within 15-30 minutes. If concentration reaches 5%, mental depression can occur within several hours [21].

In the literature, most of the studies on N95 mask use and its physiological effects on wearers have been done in the laboratory or experimental settings with the use of treadmills, bicycle ergometers, and other instruments for measurement (such as plethysmography, spirometry, capnometers, rhinomanometry, etc.).

Our study was unique as there are very few studies that show the impact on the vitals of healthcare workers due to the prolonged use of N95 masks in hospital settings. We found a similar study done by Choudhury et al. [22]. In a study done by Sanri et al., they found that end-tidal carbon dioxide, mean arterial pressure, and dyspnea scores were significantly higher in volunteers using medical masks with FFP2 versus those wearing medical masks only, although clinical implications of these changes were not observed [23].

We conducted a single-center study, so the findings cannot be generalized. A larger sample size and varying durations may be considered for future studies. A noteworthy suggestion is that infection control procedures and appropriate processes for disinfecting, changing, and maintaining respirators would need to be considered in the case of extended use of N95 masks. As this was an observational study, the partial pressures of carbon dioxide, oxygen, and lactate levels were not measured, which could have provided more conclusive evidence for the physiological changes that occur. Our study included observed or recorded values or changes, not measured ones. Another limitation of this study was the use of N95 masks, which included both with and without expiration valves, which might have had an impact on the results due to the time-to-time availability of types of N95 masks as government procurements go through certain set rules.

The concern about certain subgroups, such as pregnant healthcare workers and those with significant cardiorespiratory illnesses or COPD, may be important due to their impaired cardiorespiratory capacity. In one study done among pregnant healthcare workers using an N95 mask, there was no drive to increase breathing frequency and very minimal change in oxygen saturation [15]. Kyung et al. evaluated that people with COPD should be careful when using N95 masks as they may increase the risk of dyspnea and breathing discomfort [24]. Future studies in this subgroup can be suitably planned with strict monitoring of vitals in laboratory settings first, preferably with adequate temperature and humidity control to avoid discomfort. Once significant concerns about the severe deterioration of vitals are ruled out, studies on these populations can be carried out in hospital settings. As our study was done in hospital settings and not in a controlled environment, we excluded these subgroups from the study.

## Conclusions

Our study found a statistically non-significant mild increase in respiratory rate among healthcare workers at one, three, and six hours of continuous N95 mask use. The heart rate and oxygen saturation showed significant variations at different points in time. However, the clinical implications of these changes were largely uncertain. Thus, the participants tolerated the N95 masks well during their shifts, either clinically or in terms of perceived subjective discomfort, as the changes in vitals were statistically more significant than clinical. Observations of larger studies in a controlled environment can help to make policy decisions regarding the long-shift duties of healthcare workers wearing N95 masks, especially those working in highly infectious wards or intensive care units. An improved mask design with a focus on safety, comfort, and tolerability may possibly be further explored with a larger sample size in a controlled environment.
